# Control Models and Spatiotemporal Characteristics of Air Pollution in the Rapidly Developing Urban Agglomerations

**DOI:** 10.3390/ijerph18116177

**Published:** 2021-06-07

**Authors:** Longwu Liang, Zhenbo Wang

**Affiliations:** 1 Institute of Geographic Sciences and Natural Resources Research, Chinese Academy of Sciences, Beijing 100101, China; lianglw.17s@igsnrr.ac.cn; 2 Key Laboratory of Regional Sustainable Development Modeling, Chinese Academy of Sciences, Beijing 100101, China; 3 College of Resource and Environment, University of Chinese Academy of Sciences, Beijing 100049, China

**Keywords:** control models, spatiotemporal characteristics, air pollution, urban agglomeration, China

## Abstract

This paper systematically summarizes the hierarchical cross-regional multi-directional linkage in terms of air pollution control models implemented in the Beijing-Tianjin-Hebei urban agglomeration, including the hierarchical linkage structure of national-urban agglomeration-city, the cross-regional linkage governance of multiple provinces and municipalities, the multi-directional linkage mechanism mainly involving industry access, energy structure, green transportation, cross-regional assistance, monitoring and warning, consultation, and accountability. The concentration data of six air pollutants were used to analyze spatiotemporal characteristics. The concentrations of SO_2_, NO_2_, PM_10_, PM_2.5_, CO decreased, and the concentration of O_3_ increased from 2014 to 2017; the air pollution control has achieved good effect. The concentration of O_3_ was the highest in summer and lowest in winter, while those of other pollutants were the highest in winter and lowest in summer. The high pollution ranges of O_3_ diffused from south to north, and those of other pollutants decreased significantly from north to south. Finally, we suggest strengthening the traceability and process research of heavy pollution, increasing the traceability and process research of O_3_ pollution, promoting the joint legislation of different regions in urban agglomeration, create innovative pollution discharge supervision mechanisms, in order to provide significant reference for the joint prevention and control of air pollution in urban agglomerations.

## 1. Introduction

Rapid economic development and urbanization have concurrently boosted energy consumption and pollutant emissions while promoting nations’ overall power and social progress [1], which has led to increasingly serious environmental pollution [2] and health problems [3] in urban areas. As Samet [4] suggested, urban air pollution could become a public health and environmental problem of crisis proportions in the near future, if it is not so already. Since 2012, haze pollution has become a severe environmental problem that has impacted individuals’ health and daily lives in China [5,6]. On the one hand, the continuous spread of air pollution on a large scale endangers the health of local residents, especially the elderly [7]. On the other hand, pollution has huge economic consequences. China will lose 2% of its total GDP and bear healthcare costs of USD 25.2 billion until 2030 [8]. As one of the five national-level urban agglomerations in China, the Beijing–Tianjin–Hebei urban agglomeration (BTHUA) is the implementation of the regional coordinated development strategy, and it is also the region with the most serious air pollution [9,10] and the most acute contradiction between resources, environment, and development [11] in China. Eight of the ten cities with the highest average annual concentration of PM_2.5_ were located in BTHUA in 2015 [9]. Therefore, the status of high strategic status, high population density, high development speed, high environmental pollution, high energy emissions, and low environmental carrying capacity has led to the great conflict between regional economic development and environmental protection in BTHUA [12]. The central and regional governments have put forward many policies, models, and strategies for the air pollution control in BTHUA from the scale of nation, urban agglomeration, city, and county. It is urgent to systematically sort out and summarize them, so as to provide significant reference for the prevention and control of air pollution in other regions.

Air pollution is the result of the comprehensive effects of natural climate, topography, ecological environment, economic, and social factors in urban areas, determining the long-term, arduous, and complex prevention and control of air pollution. In recent years, scholars from all countries have created rich academic literature on air pollution chemical composition [13], spatiotemporal characteristics [14], source apportionment [15], impact mechanism [16], prevention and control model [17], regional division of joint control [18], and other related topics. Specifically, the influencing factors of air pollution include urbanization level [19], energy consumption structure [20], transportation infrastructure construction [21], foreign direct investment [22], climate conditions [23], policy elements [24], etc. In the international literature of urban air pollution, Liu et al. [25] found that the atmospheric pollutants revealed a stable trend from 2008 to 2018 in Germany. Hossain et al. [26] found that the emission control measures resulted in NO_2_, SO_2_, and PM reductions from 2000 to 2018. Chakraborty and Basu [27] considered that PM_2.5_ concentration levels were significantly higher in more urbanized districts located predominantly in northern India. Practical experience shows that developing strict air quality standards and pollution control policies through cross-regional governance agencies, industrial restructuring, using clean energy and renewable energy, implementing and improving public transport, planning urban infrastructure, and pricing electricity for more efficient usage can effectively reduce air pollution [28]. In the short term, realistic approaches include energy conservation, promoting mass transit, reducing open burning, instituting motor vehicle inspection programs, and phasing out lead in petrol. In the longer term, air quality management strategies, such as urban and transportation planning, institutional and technological limitations should be incorporated in industrial and urban development [29].

In order to improve the air quality, the Chinese government has made great efforts in the control of air pollution, the ultra-low emission policy of China’s thermal power industry has led to a significant reduction in the emissions of various air pollutants [30]. As the leading area of joint prevention and control models of air pollution in China, the air pollution control in the BTHUA has become a benchmark for measuring the effectiveness of urban environmental governance in China. However, there are few studies systematically sorting out and summarizing the air pollution control models from the regional perspective, and few studies have explored the spatiotemporal changes characteristics of the air pollution in BTHUA from the perspective of the air pollution prevention and control effectiveness.

Therefore, based on the air pollution control models of different cities in BTHUA, this paper forms the hierarchical cross-regional multi-directional linkage (HCML) in terms of air pollution prevention and control models, in order to reduce air pollutant concentration and adverse health effects. This paper introduces the structural characteristics and specific strategies of the models in detail from the model architecture, model management, and model guarantee. In order to explore the effectiveness of this model in air pollution control, this paper uses statistical method and spatial pattern analysis method to quantitatively analyze the annual, quarterly, and monthly and compliance rate characteristic of six air pollutants (PM_2.5_, PM_10_, SO_2_, O_3_, NO_2_, and CO) in BTHUA from 2014 to 2017. Finally, we further discussed the problems of air pollution control and the recommendations of research and policy about the efficient control.

## 2. Hierarchical Cross-Regional Multi-Directional Linkage in Terms of Air Pollution Control Models

**Figure 1:**
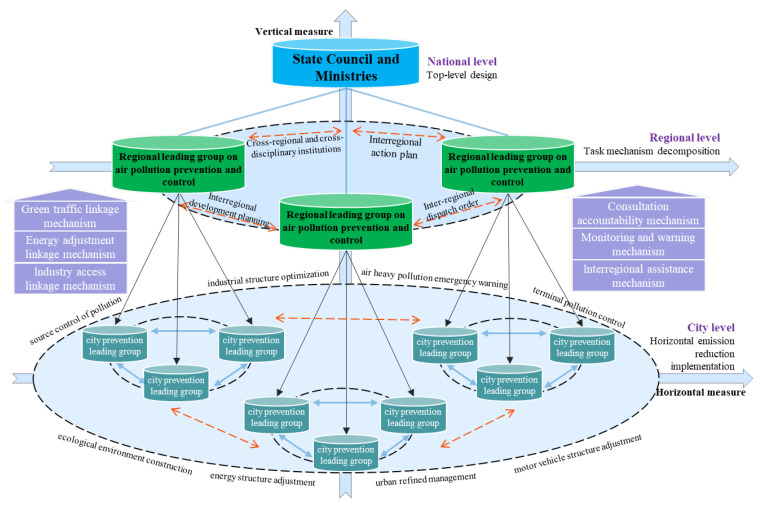
Hierarchical cross-regional multi-directional linkage model in terms of air pollution in BTHUA.

The atmosphere is a public resource that exhibits fluidity and infinity. Its state and composition are directly affected by natural geographical factors, such as solar radiation, temperature, humidity, air pressure, wind direction, precipitation, and other meteorological and topographical elements. The state and composition of the atmosphere are also indirectly affected by interactions between atmospheric particulate and pollution emissions resulting from human activity, such as the use of motor vehicles, industrial production, and coal burning. Based on the spatial and temporal attributes of pollution sources and pollution spread, the government also coordinates multiple levels of countries, urban agglomerations, cities, and enterprises and continuously adopts actions for the prevention and control of air pollution via direct and indirect governance. We summarize this action as the hierarchical cross-regional multi-directional linkage in terms of air pollution control models (Figure 1), which comprises the following framework and operational mechanism [17].

### 2.1. Linkage Architecture of Vertical Layer

First, a top-level design plan was created at the national level. In 2013, the governance issued the “Air Pollution Prevention and Control Action Plan” (hereinafter referred to as the “Plan”), proposing overall requirements, goals, and policy measures for the prevention and control of air pollution nationwide. After 2014, the relevant departments successively issued control documents, such as the “Air Pollution Prevention and Control Law”, the new “Environmental Protection Law of the People’s Republic of China”, “Urban Environmental Air Quality Change Degree Ranking Scheme”, and the “Fire Power Plant Pollution Prevention and Control Technology Policy”, to further refine the air pollution control objectives. Second, task decomposition and accountability mechanisms were implemented in the urban agglomerations. The state council authorized the Ministry of Environmental Protection and the people’s governments of all provinces (autonomous regions and municipalities) within the urban agglomeration to sign the responsibility letter for air pollution prevention and control; breakdown the emission reduction tasks to all levels of government, departments, and enterprises; and establish the air pollution control responsibility assessment system both vertically and laterally. Third, specific plans were implemented for lateral emission reductions at the city level. A city project management system of energy conservation and emission reduction was established, including source control, end pollution control, industrial structure optimization, energy structure upgraded, motor vehicle structure adjustment, ecological environment construction, and air heavy pollution emergency prevention and control projects. Finally, a “special responsibility list” was developed, the territorial management responsibilities of the governments at all levels determined, and the responsibilities divided between various departments in the city.

### 2.2. Linkage Governance of Horizontal Cross-Region

First, the air pollution control agency was established cross-regionally. In 2013, air pollution prevention and control cooperation groups were established in BTHUA and the surrounding area. In response to the pollution problem, regular meetings were organized to engage the government official for the provinces and cities, such as Beijing, Tianjin, Hebei, Shanxi, Shandong, Neimenggu, Henan, and other relevant ministries and commissions. Second, short-term prevention targets were identified in the interregional action plans. The “Beijing–Tianjin–Hebei Air Pollution Prevention and Control Action Plan Implementation Rules” (hereinafter referred to as the “Rules”), jointly issued by the six ministries and commissions, such as the Ministry of Environmental Protection, clearly defined the annual average concentration control targets for particulate matter and environmental standards for industrial access and elimination tasks of the high-pollution industries in provinces and cities. Third, cross-regional collaborative planning was used to develop long-term prevention and control routes. The “BTHUA Collaborative Development Plan” and the “BTHUA National Economic and Social Development Plan during the 13th Five-Year Plan” proposed the construction of an ecological restoration environment improvement demonstration zone, and defined the framework and models for pollution prevention and green development in BTHUA. Fourth, the dispatch order system was created cross-regionally. In 2016, the system was initiated and implemented in Hebei Province. The dispatching order is authorized by the provincial government to improve atmospheric environmental quality and address regionally polluted areas and outstanding environmental issues, using the results from research on pollution sources. The order can also schedule key tasks, such as the reduction of industrial enterprises, use of clean energy, motor vehicle restrictions, urban dust control, and non-point-source pollution control in specific regions.

### 2.3. Linkage Mechanism of Synergistic Multi-Direction

The industrial structure, energy structure, and transportation network pattern are the main influencing factors of air pollution in BTHUA. Based on this observation, the governments at all levels of the urban agglomeration formulated the following linkage measures.

The first is the heavy-industry access linkage mechanism. Excess and new capacity projects, such as steel, cement, electrolytic aluminum, flat glass, ship, coking, colored, calcium carbide, and ferroalloy, cannot be approved in BTHUA and its surrounding areas. General manufacturing and new capacity labor-intensive projects cannot be approved, and existing projects are gradually shifting outward from Beijing. The six major industries, thermal power, iron and steel, petrochemical, cement, nonferrous metals and chemical industries, and coal-fired boiler projects, must strictly impose special emission limits for atmospheric pollutants in the BTHUA. Concurrently, highly polluting companies’ elimination lists and capacity reduction targets were formulated in various provinces.

The second is the energy structure adjustment linkage mechanism. The government implemented a control plan for total coal consumption, reducing total consumption by 83 million tons in 2017; implemented a clean energy replacement plan; increased the proportion of clean energy use; promoted a coal clean utilization plan; expanded the scope of high-pollution fuel-free zones; promoted efficient and clean heating and solar water heating systems; optimized industrial space layout; and completed the relocation and transformation of heavy-polluting enterprises, such as steel, petrochemical, and chemical industries, in the main urban area. As a result of these measures, coal accounted for less than 10% of energy production in Beijing.

The third is the green transportation linkage mechanism. The government optimized the intercity integrated transportation system for the urban agglomerations; implemented a bus priority strategy; increased the proportion of green transportation; increased the proportion of public transportation by more than 60% in Beijing and Tianjin in 2017; controlled the number of motor vehicles, simultaneously implemented total motor vehicle control; limited travel time according to license plate tail number in Beijing, Tianjin, and Langfang; eliminated all yellow label cars (those heavy-polluting vehicles) at the end of 2017; upgraded the fuel quality to meet the national fifth stage standard; promoted new energy vehicles; and increased the proportion of new energy and clean fuel vehicles to 60% in Beijing, Tianjin, and the provincial capital cities.

The fourth is the inter-regional assistance linkage mechanism. Simultaneously with the implementation of the regional internal linkage plan, the joint pollution control programs of Beijing and Tianjin assisting the key polluted cities in Hebei Province were executed. Beijing provided 460 million yuan to compensate Langfang and Baoding for joint pollution control activities, with reducing the coal-fired emissions by 0.770 million tons and the annual sulfur dioxide emissions by more than 6 thousand tons in 2014. Tianjin invested 400 million yuan in special funds to support air pollution control projects in Zhangzhou and Tangshan in 2016.

The fifth is the monitoring and early warning linkage mechanism. The government established a unified national air quality monitoring network in prefecture-level cities, improved the online monitoring system for pollution sources, built a monitoring platform for motor vehicle sewage discharge, established a monitoring and early warning system for heavy-pollution weather, prepared heavy-pollution emergency plans, and implemented a regional emergency response mechanism for heavy-pollution weather.

The sixth is the consultation and accountability mechanism. The provincial, municipality, and autonomous region governments and relevant ministries and commissions jointly coordinated efforts to address major environmental issues as well as to organize and implement environmental assessments, information sharing, joint law enforcement, early warning, and emergency measures. The state Council, provinces, and cities signed the responsibility book for air pollution prevention and control objectives, with each organization respectively implementing their pollution control task. The assessment system was established by focusing on government assessment whilst considering third-party input. Pollution control status reports from previous years are assessed in a timely manner, and the results reported to the state council and announced to the public.

## 3. Data Methods and Framework

### 3.1. Data Source and Initial Processing

**Figure 2:**
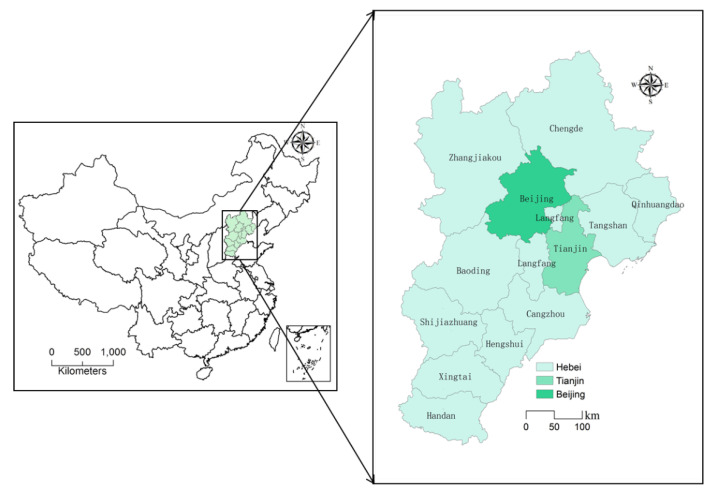
Location of the study area in China.

The research scopes are the 13 cities above the prefecture level in the BTHUA, including Beijing, Tianjin, Shijiazhuang, Baoding, Zhangzhou, Chengde, Handan, Hengshui, Langfang, Qinhuangdao, Tangshan, Xingtai, and Zhangjiakou; these cities cover an area of 0.22 million km^2^ (Figure 2). In 2017, the BTHUA carried 8% of the country’s population and contributed 10% of the country’s total economic output with a national land area of 2.3%. Data for SO_2_, NO_2_, PM_10_, PM_2.5_, O_3_, and CO concentration were obtained from the urban air quality real-time release platform of the China Environmental Monitoring Station. According to the effectiveness requirements of air pollutant concentration data in the GB3095-2012 [31], with reference to the research results [6], the quality of monitoring data was controlled. Because the number of urban inspection points and daily data time points are different, this paper averaged the data to obtain mean daily concentration values for the six pollutants. Daily average refers to the arithmetic mean of the 24-h average concentration of a natural day, monthly average refers to the arithmetic mean of the average daily concentration in a calendar month, quarter average refers to the average concentration for each day in a calendar season, and annual average refers to the arithmetic mean of the average concentration of each day in a calendar year. In addition, spring season refers to the months of March–May, summer to June–August, autumn to September–November, and winter to December–February.

### 3.2. Spatial Pattern Analysis Method

Spatial interpolation is a method of estimating unknown points by using locally known points according to the correlation of adjacent regions in geospatial space. The surface is created by a highly accurate interpolation method with known sample selection. The spatial analysis of air pollution mainly uses kriging interpolation method, inverse distance weight interpolation, geographic weighted regression model [32], and geographic semivariogram method [33] to estimate spatial differentiation. This study uses the geostatistical wizard of geostatistical analyst in ArcGIS software to perform spatial interpolation and to accurately analyze the spatial distribution of six pollutants in the BTHUA. The commonly used methods mainly include inverse distance weighted, spline method, and kriging method. The geostatistical kriging interpolation method is the most flexible, and the result is the highest precision [34]. The formula is as follows:

(1) $$h\left(x_{0}\right)=\overset{n}{\underset{i=1}{\sum }}\lambda_{i}h\left(x_{i}\right)$$

Among them, $h\left(x_{0}\right)$ is the monitoring value of the point $x_{0}$; $h\left(x_{i}\right)$ is the monitoring value of the point $x_{i}$; $\lambda_{i}$ is the kriging weight coefficient; n is the total number of monitored cities. For the selection of different semivariograms (exponential model, triangular model, spherical model, Gaussian model, linear model) in kriging method, this study used the cross-validation of spatial interpolation results to compare the average absolute error and the root mean square error, and finally selected the higher precision function model as the method for analyzing the spatial pattern of atmospheric pollution in the BTHUA.

### 3.3. Research Framework

**Figure 3:**
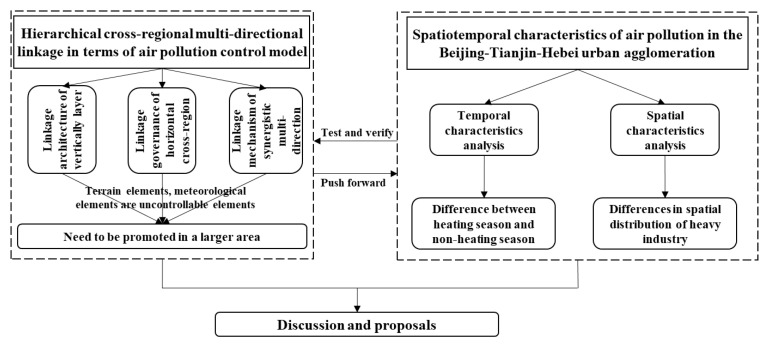
The logical framework of this study.

Air pollution has significant spatial and temporal differentiation in BTHUA. The fundamental reason is that its topography, industry, meteorology, and urban scale have significant spatial differentiation. The differentiation of time is reflected in the difference between heating season and non-heating season. The phenomenon of “scattering pollution” in Baoding and other cities is serious, and the amount of loose coal is large and the emission level is high. The differentiation of space is mainly determined by the spatial distribution of high-emission and high-pollution heavy industry. The total emissions and intensity are large with the steel, glass, petrochemical, chemical industry, and the supporting heavy transport vehicles in Tangshan, Tianjin, Shijiazhuang, Handan, and Xingtai. The main goal of the model governance is to prevent and control air pollution by adjusting the industrial structure, energy structure, transportation structure, land use structure, and emergency measures in BTHUA. However, topographical and meteorological elements are uncontrollable, and the government cannot regulate the flow of pollutants from outside the BTHUA into the area. Therefore, this model needs to be promoted in a larger area, even nationally and internationally. Based on the above analysis, this paper made the following logical framework diagram (Figure 3).

## 4. Spatiotemporal Characteristics of Air Pollution in the BTHUA

This section uses the kriging interpolation method to analyze the timing evolution and the spatial pattern characteristics of air pollution from 2014 to 2017 in BTHUA, respectively. The thorough comparison of 112 ground monitoring stations, primarily across the BTHUA, was conducted to evaluate the concentrations of the main atmospheric pollutants from 2014 to 2017.

### 4.1. Air Pollution Concentration Analysis

**Figure 4:**
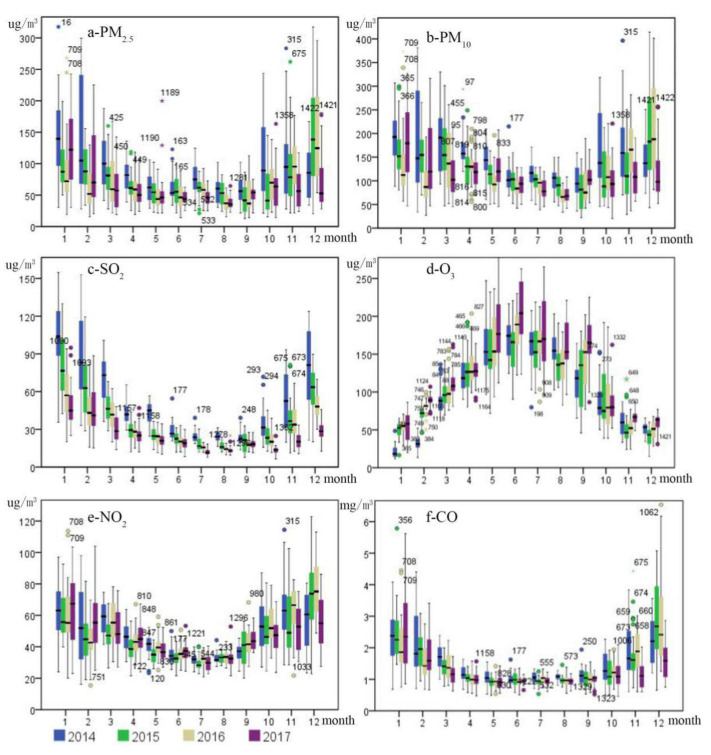
Trends in the seasonal variations of six air pollutant concentrations in BTHUA from 2014 to 2017.

Air quality significantly improved from 2014 to 2017 in the BTHUA. Except for O_3_, the concentration of other air pollutants declined, the high-pollution spatial scope decreased from north to south. The mean annual concentrations of PM_2.5_, PM_10_, SO_2_, NO_2_, and CO decreased by 30%, 23%, 52%, 3%, and 17%, respectively. Therefore, a large overall improvement of air quality was achieved, confirming the successful implementation of air pollution controls (Figure 4); however, the overall concentration value is still high, and the decline range is far from reaching the environmental capacity, so the task of air pollution prevention and control is still arduous. From a year-on-year comparison, the concentrations of PM_2.5_, PM_10_, SO_2_, and CO continued to decline from 2014 to 2017, but the downward trend slowed down, indicating that the difficulty of air pollution control increased with the overall improvement of air quality [35]. The concentration of NO_2_ showed a downward trend as a whole but increased in 2016, indicating that the effect of air pollution control has certain volatility [36]. The concentration of O_3_ continues to rise, and the rising trend is more significant, which indicates that the problem of O_3_ pollution has become more and more serious in recent years, which needs to arouse the attention of relevant government departments. At the same time, it also needs the academic community to carry out in-depth research on the chemical composition, impact mechanism, and prevention and control models of O_3_ in urban agglomerations [37].

In terms of the monthly change trend, the concentrations of PM_2.5_, PM_10_, and SO_2_ kept a downward trend year by year from 2014 to 2017, and the prevention and control measures were significantly effective. The concentrations of NO_2_ and CO showed a trend of first rising and then decreasing, it is still necessary to further strengthen pollution prevention and control; the concentration of O_3_ kept increasing year by year on the whole. Therefore, it is necessary to attach great importance to O_3_ pollution and strictly implement an ultra-low emission policy [38]. Specifically, compared with the monthly pollutant concentration in 2014, the concentrations of PM_2.5_ and SO_2_ decreased significantly in all months in 2017, the concentrations of PM_10_ and CO increased only in January, and decreased significantly in other months; the concentration of NO_2_ increased in January, February, June, September, December, and decreased in other months; the concentration of O_3_ only decreased in October and increased significantly in other months.

In terms of the seasonal average concentration, the concentrations of PM_2.5_, NO_2_, CO, PM_10_, and SO_2_ were the highest in winter, the lowest in summer, the middle in spring and autumn, the concentrations of the first three were higher in autumn than in spring, and the concentrations of the last two were higher in spring than in autumn. The coal burning and heavy industrial production increased the emission of the above pollutants [39], the adverse climate conditions led to the increase of PM_2.5_ concentrations [40]. The concentration of O_3_ was the highest in summer, the lowest in winter, and medium in spring and autumn. Under strong sunlight, nitrogen oxides and volatile organic compounds (VOCs) can produce O_3_ by photochemical reaction [41]. In terms of the seasonal change trend, compared with that in 2014, the concentrations of PM_2.5_, PM_10_, SO_2_, and CO showed a downward trend in all quarters in 2017, the concentration of PM_10_ decreased significantly, followed by PM_2.5_. The concentration of NO_2_ only increased by 1.5% in winter, the other quarters showed a downward trend [42]. The concentration of O_3_ showed an upward trend in all quarters. The reduction degrees of pollutant concentration in autumn and winter were higher than those in spring and autumn, and the government effects were more significant in autumn and winter.

### 4.2. Analysis of Pollutant Compliance Rate

**Figure 5:**
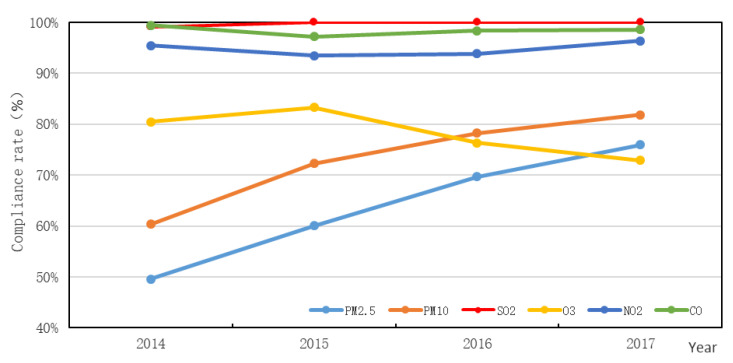
Trends in compliance rates for six air pollutants from 2014 to 2017.

According to the requirements of GB 3095-2012, the 24 h average concentration limits of a second-class region for PM_2.5_, PM_10_, SO_2_, O_3_, NO_2_, and CO are 75 μg/m^3^, 150 μg/m^3^, 150 μg/m^3^, 160 μg/m^3^, 80 μg/m^3^, and 4 mg/m^3^, respectively. Excluding O_3_ and CO, the concentrations of the other air pollutants showed a significant growth in the rate of compliance in the BTHUA from 2014 to 2017 (Figure 5). The compliance rates of PM_2.5_, PM_10_, and SO_2_ continued to increase by 25.6%, 21.50%, 0.85%, respectively; PM_2.5_ increased from 49.58% to 75.98%; PM_10_ increased from 60.34% to 81.84%; SO_2_ increased from 99.15% to 100%. The compliance rate of NO_2_ first decreased and then increased, with an overall increase of 0.9%, 95.47% in 2014; 93.48% in 2015; and 96.37% in 2017. The compliance rate of CO also first declined and then rose, with an overall decline of 0.83%, 99.43% in 2014; 97.17% in 2015; and 98.60% in 2017. The compliance rate of O_3_ first increased and then decreased, with an overall decrease of 7.54%, 80.45% in 2014; 83.29% in 2015; and 72.91% in 2017.

### 4.3. Spatial Characteristics of Air Pollution

**Figure 6:**
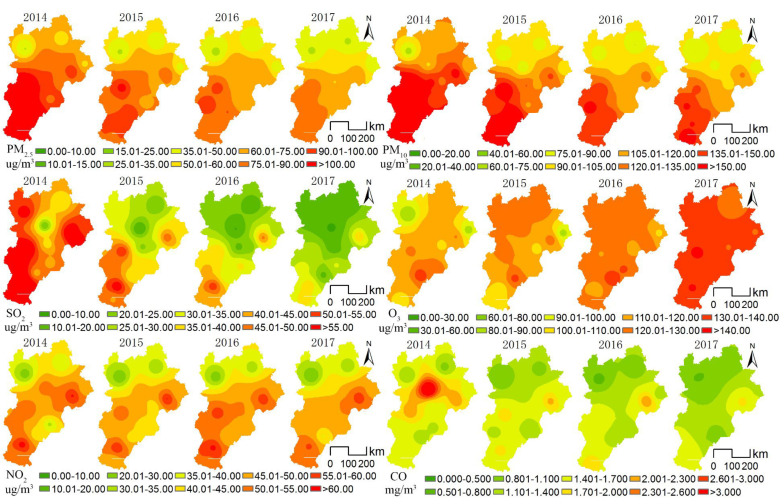
Spatial pattern of annual mean values of six air pollutants in BTHUA from 2014 to 2017.

The paper uses kriging interpolation method in the ArcGIS software to perform spatial interpolation analysis on the site data of six atmospheric pollutants in the BTHUA. The air quality high-pollution range decreased significantly in the BTHUA from 2014 to 2017, excluding that the O_3_ high-pollution range spread from south to north, the high pollution ranges for other pollutant decreased significantly from north to south in the BTHUA (Figure 6). Concentrations of PM_2.5_ and PM_10_ both showed a pattern of high values in the south and low values in the north, and the concentrations decreased in the high-polluted southern areas more than those in the low-polluted northern areas. Pollution in excess of the 75 μg/m^3^ standard line for PM_2.5_ concentration retreated to the south of Beijing and Tianjin, but the PM_10_ concentration in the entire urban agglomeration was higher than the national annual limit of 70 μg/m^3^. Areas with large fluctuations in PM_2.5_ concentrations are mainly concentrated in the central of urban agglomeration, such as Beijing, Tianjin, Hengshui, Langfang, and Baoding, indicating that it is more difficult to control the PM_2.5_ pollution in the central area of the urban agglomeration with Beijing–Tianjin as the core. SO_2_ concentrations show a spatial pattern of high values in the south and low values in the north, these values decreased significantly in the four quarters, the decline in the north was more significant than that in the south, with the concentrations decreasing significantly in Xingtai, Baoding, Shijiazhuang, and Tangshan. The only pollutant with an increased concentration was O_3_ in the urban agglomeration, the average annual concentration increased by 21 μg/m^3^ (up 20.59%) in four years [43]. The O_3_ concentrations decreased in the south of Beijing, such as Zhangzhou, Xingtai, Handan, Hengshui, Tianjin, and Shijiazhuang, increased in the north, with those increasing by 75% in Zhangjiakou. NO_2_ concentrations declined first, then increased in 2016, and declined again in 2017, with an overall decline of 3% over four years. Cangzhou and Hengshui have become the pollution centers in the south, while other regions have shown a downward trend. Although the concentrations in Tangshan have decreased, it is still a northern pollution center due to the high concentration. The concentration of CO decreased by 17% in four years, with decreases in the central cities of Beijing, Tianjin, Zhangjiakou, and Baoding driving the overall concentration in the urban agglomeration, which decreased by 33% and 18% in Beijing and Tianjin, respectively, the concentration increased in the south, i.e., in Hengshui, Chengde, Langfang, and Xingtai.

## 5. Discussion and Proposals

### 5.1. Discussion on the Air Pollution Control Models in China

The implementation of the HCML model has effectively improved the air quality in heavily polluted areas over a short time period in China. On the whole, China’s prevention and control strategies have the following unique characteristics. First, it has the most stringent laws in China, such as the Environmental Protection Law of the People’s Republic of China (Revised in 2014) and Atmospheric Pollution Prevention and Control Law of the People’s Republic of China (2015 Revision). Companies are subject to more stringent standards, with harsher penalties. Illegal sewage discharge from illegal enterprises is punished on a daily basis, and penalties are not capped. Furthermore, the four types of non-crime, such as the implementation of environmental impact assessment, are subject to administrative detainment. The local government is responsible for the quality of the environment in its jurisdiction, and the environmental protection objectives are linked to the administrative personnel evaluations. The government controls the total amount of pollution discharge and limits the projects that exceed the total amount in the region. Regulatory authorities have also been endowed more responsibility: the environmental protection department has greater power to directly shut down illegal enterprises, and relevant unregulated officials have strict administrative accountability.

Second, the model has the toughest plan to date for combating air pollution, the Air Pollution Prevention and Control Action Plan. This plan is the largest governance effort, with the tightest protection measures and most stringent assessment. For the first time, the action plan incorporates fine particulate matter into binding indicators and incorporates environmental quality improvement into the official assessment system. The state council and provincial (district, municipal) people’s governments sign the responsibility book for air pollution prevention and control targets and breakdown the target tasks for distribution to the local people’s government. Enterprises adopt administrative means to force the completion of capacity-reduction plans for 21 key industries, including steel, cement, electrolytic aluminum, and flat glass enterprises, according to the timetable.

Third, the model provides a fully covered weather monitoring and early warning system and the national–provincial–city–enterprise linkage action mechanism. In 2015, 31 provinces (autonomous regions and municipalities) implemented provincial air quality monitoring and early warning systems in China, and 32 cities and provincial capital cities launched municipal air quality monitoring and early warning systems. Aiming at the spatiotemporal characteristics of air pollution, the national–provincial–municipal–department–enterprise management structure was constructed, including the target decomposition models and the fast dispatching and execution models.

### 5.2. Discussion of Research Proposals

#### 5.2.1. Traceability and Process Research of Heavy Pollution in Autumn and Winter

According to the trend analysis, air pollution is reduced significantly from the spring to the summer of the subsequent year and increased in the autumn and winter in BTHUA. The volatility results showed that the daily average concentration volatility of pollutants showed a weakening trend and tended to be stable, but the time performance was lower in the first half of the year and higher in the second half. Therefore, air pollution in autumn and winter is the focus of future prevention and control, especially in winter, which is the season in which the most serious air pollution occurs in the BTHUA. Using PM_2.5_ as an example, in 2014 and 2015, the proportion of winter pollution days was more than 70% (60 days), accounting for 35% and 43% of the total number of those days in the year, respectively, and the number of days with heavy pollution accounted for 54% and 83% of the total number of those days in the year, respectively. Therefore, it is critical to strengthen the traceability and process research of heavy pollution in the winter coal burning period [44].

#### 5.2.2. Traceability and Process Research of O_3_ Pollution

Ozone is the main component of smog in Los Angeles; its formation is directly related to automobile exhaust and gas particle conversion due to photochemical reactions. It is also one of the most prominent air pollution problems in the Beijing–Tianjin–Hebei urban agglomeration [45]. Among the six pollutants in the BTHUA, only the levels of O_3_ pollution are increasing, and the increase is more significant in the summer in the southern region with Hengshui as the general center. Pan et al. [46] proposed that the increase in the relative contribution of nitrate to PM_1_ observed during the early stages of haze pollution was due to new particle formation, whereas the nitrate formed in PM_1–2.5_ during the latter stages was due to heterogeneous formation and hygroscopic growth. Therefore, controlling NOx emissions should be a priority for improving air quality in mega cities of the BTHUA [47]. The BTHUA is located in the VOC control area, and VOC emissions have not been effectively controlled, resulting in an upward trend of O_3_ and PM_2.5_ pollution. The VOC emission reduction project should be implemented to control O_3_ and PM_2.5_ emissions [48]. Given the health hazards, the explanations, processes, and solutions for aggravated O_3_ pollution in the BTHUA urgently warrant further research.

### 5.3. Policy Recommendations

#### 5.3.1. Multi-Regionally Joint Legislation and Implementing Policies 

There are significant spatial differences in the high-pollution range in BTHUA, the air pollution was serious with dense population and rapid industrial development in the central and southern region. However, the intensity of fighting against pollution is weak, and the legal treatment awareness of pollution is not strong in those areas; it is urgent to carry out coordinated legislation and multi-regional linkage measures and strengthen market-oriented policies and incentive measures in BTHUA [49]. Taking cues from the implementation of the new “Air Pollution Prevention and Control Law” and the “Environmental Protection Law”, a coordinated legislative mechanism that is compatible with the coordinated development of the BTHUA may be developed. This mechanism will be mutually supportive and reinforcing and enable the development of coordinated legislation in key areas by integrating and matching relevant regulations from different locations, continuing to refine legal constraints, eliminating administrative barriers and local protection through legislation, and ensuring that all localities can conduct concerted and precise attacks on air-polluting behavior.

#### 5.3.2. Innovative Pollution Discharge Supervision Mechanisms and Public Supervision 

In recent years, the air quality has been improved, and the pollution control has certain effect, but it has obvious volatility; the air pollution problem is still relatively serious in BTHUA. Industrial pollution, motor vehicle pollution, and heating coal in winter are the main reasons for the air pollution of urban agglomeration. With people’s desire for a better life, public participation is very important in air pollution control. Therefore, it is necessary to further innovate the emission supervision mode and improve the public supervision mechanism [50]. Both the “plan” and the “details” clarify the objectives for the closure and renovation of industrial enterprises and key industries in the BTHUA. However, the number of industrial enterprises within the urban agglomeration is huge. The proportion of small- and medium-size enterprises is over 96%, and their locations are scattered. There are many issues, such as undocumented smuggling, night smuggling, online data fraud, and no using pollution control facilities. Concurrently, the number of management supervisors cannot meet the need of enterprises, which, in turn, impacts the regulation of atmospheric pollutant discharge. Therefore, the implementation of a unified supervision mechanism for multi-sectors and multi-subjects and a joint supervision model for the implementation of responsibilities and improved public supervision are inevitable directions for pollution supervision work.

## 6. Conclusions

Air pollution caused by the rapid development of Chinese cities in the past few decades and its impact on resident health cannot be ignored [51]. The governments of the BTHUA have achieved certain progress in implementing the “stratified cross-region multi-directional linkage” air pollution prevention and control model. However, the industrial structure, energy structure, and traffic pattern in the current industrialization stage continues to pose long-term challenges to air pollution prevention and control measures in urban agglomerations.

Based on air pollution control in BTHUA, this paper constructs the hierarchical cross-regional multi-directional linkage in terms of air pollution control models to provide significant reference and theoretical support for air pollution control in other regions of China and other countries in the world. We hope to arouse the discussion and application of this models and the practical details and problems in the academic societies. However, we do not further reveal the issue of policy transferability by using the quantitative methods, which is the focus of our future research. In addition, each region and each country have different difficulties for air pollution control. Therefore, it is necessary to consider the specific characteristics of the city when controlling the air pollution.

From the perspective of the effectiveness of air pollution control, this paper explores the change characteristics of six pollutants, namely, SO_2_, NO_2_, PM_10_, PM_2.5_, O_3_, and CO in BTHUA from 2014 to 2017. Due to the length of the article, we do not further reveal the impact mechanism of different air pollutants. Impact mechanism and traceability analysis is important for the radical cure of air pollution, which is a meaningful direction of our future research. In the spatial analysis section of this paper, we use the statistical cokriging interpolation method of statistics, which is the spatial analysis method used in geography. We can also use other advanced methods to explore the spatial characteristics of air pollutants.

## Data Availability

Data are available upon request.
